# Chemical stress in a largely deformed electrode: Effects of trapping lithium

**DOI:** 10.1016/j.isci.2023.108174

**Published:** 2023-10-10

**Authors:** Yong Li, He Huang, Kai Zhang, Mi Hou, Fuqian Yang

**Affiliations:** 1 School of Intelligent Manufacturing and Control Engineering, Shanghai Polytechnic University, Shanghai 201209, China; 2 Jiangsu Key Laboratory of Engineering Mechanics, School of Civil Engineering, Southeast University, Nanjing, Jiangsu 210096, China; 3 School of Aerospace Engineering and Applied Mechanics, Tongji University, No.1239 Siping Road, Shanghai 200092, China; 4 Shanghai Pudong Software Technologies Services Co., Ltd., No. 498 Guoshoujin Road, Shanghai 201203, China; 5 Materials Program, Department of Chemical and Materials Engineering, University of Kentucky, Lexington, KY 40506, USA

**Keywords:** Electrochemistry, Electrochemical energy storage, Electrochemical energy conversion

## Abstract

Lithium trapping, which is associated with the immobilization of lithium and is one of key factors contributing to structural degradation of lithium-ion batteries during electrochemical cycling, can exacerbate mechanical stress and ultimately cause the capacity loss and battery failure. Currently, there are few studies focusing on how lithium trapping contributes to mechanical stress during electrochemical cycling. This study incorporates the contribution of lithium trapping in the analysis of mechanical stress and mass transport in the framework of finite deformation. Two de-lithiation scenarios are analyzed: one with a constant concentration of trapped lithium and the other with inhomogeneous distribution of trapped lithium. The results show that the constant concentration of trapped lithium increases chemical stress and the inhomogeneous distribution of trapped lithium causes the decrease of chemical stress. The findings can serve as a basis for developing effective strategies to mitigate the lithium trapping and improve the battery performance.

## Highlights

• Propose a chemo-mechanical model with contribution of trapping lithium
• Both mobile and immobile lithium contribute to lithiation-induced eigen-strain
• Nonuniform trapped lithium reduces the concentration gradient of resultant lithium

## Graphical abstract

**Figure:**
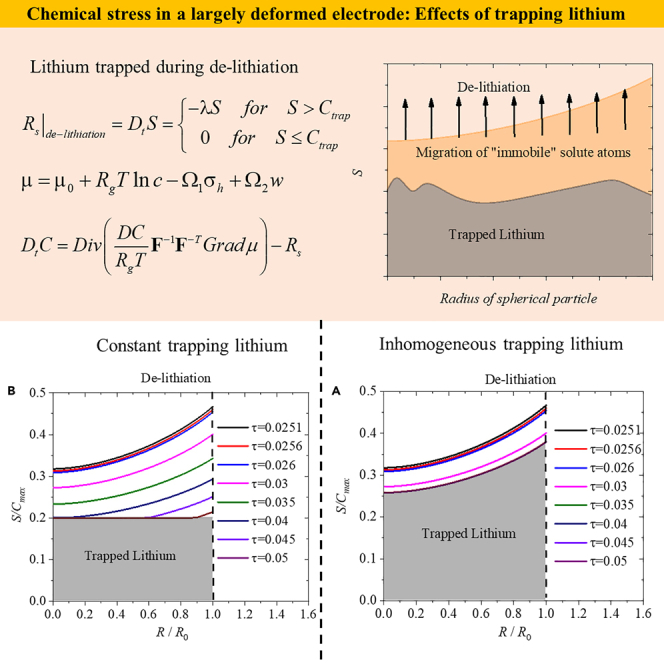

## Introduction

Clean energy has become increasingly important in today’s world due to the growing concern over climate change and the adverse effects of fossil fuels on the environment.^1^^,^^2^^,^^3^ Renewable energies, including solar energy and wind power, have gained significant interests as they offer sustainable alternatives to fossil fuels.^4^^,^^5^^,^^6^ Lithium-ion batteries play a crucial role in promoting clean energy and are widely utilized in electric vehicles and storage systems of renewable energies.^7^^,^^8^^,^^9^ However, diffusion-induced stress (chemical stress) in lithium-ion batteries, which has emerged as a pertinent issue in recent years,^10^^,^^11^ can expedite the degradation of batteries, thereby reducing their service life and posing safety risks. Developing strategies to mitigate the diffusion-induced stress in lithium-ion batteries is essential to ensure their reliability and durability in supporting the adoption of clean energy technologies.^12^^,^^13^^,^^14^^,^^15^

Lithium trapping occurs when a subset of the lithium that is inserted into active materials during lithiation becomes immobile due to solid state reactions and is unable to diffuse out of electrode during de-lithiation.^16^^,^^17^ The trapped lithium can result in an overcharge of electrode, likely leading to an increase in chemical stress and exacerbating structural degradation.^18^^,^^19^ As more lithium becomes trapped, the overall performance of a battery deteriorates, which can lead to the loss of capacity and the decrease of lifespan and eventually to the failure of the battery.^20^ Several strategies have been explored to mitigate the lithium trapping, such as forming protective coatings,^21^ increasing the conductivity of electrolyte,^22^^,^^23^ and modifying the electrode design.^24^ Zhou et al.^25^ found that the nanopores formed inside anode materials, such as silicon, germanium, and tin, during electrochemical cycling likely are the sites for the trapping and accumulation of lithium.

Along with experimental study of lithium-ion batteries, modeling and simulation have been used to investigate chemo-mechanical behavior presented in lithium-ion batteries and proven to be an effective way in understanding the evolution of chemical stress and mass transport in electrode during lithiation and de-lithiation.^26^^,^^27^^,^^28^ Wu et al.^29^ utilized a flexible sigmoid function to model the two-phase lithiation process in silicon electrodes and demonstrated the feasibility in determining the critical thickness of the lithiated phase. Singh and Bhandakkar^30^ introduced a semi-analytical approach to investigate the deformation behavior of a cylindrical, linear elastic electrode particle embedded in an annular, linear viscoelastic binder under lithiation. Christensen^31^ incorporated Dualfiol lithium-ion cell-sandwich model and a thermal analogy method in analyzing diffusion-induced stress in spherical lithium-ion activate materials with porous structures. Based on Gibbs free energy, Ji et al.^32^ developed a multiscale model that considered diffusion-induced stress and polydispersity of electrode particle sizes in porous battery electrodes. Liu et al.^33^ examined diffusion-induced stresses in a bilayer electrode with the Halpin-Tsai model and the rule of mixture for the material properties. Yang et al.^28^ developed a multiplicative finite strain deformation theory to analyze diffusion-induced stress in largely deformed electrodes. Their study is based on the change rate of the volume of the host material as a parameter describing diffusion-induced volumetric expansion. Shi et al.^34^ developed a coupled semi-analytical model to investigate mechanical-electrochemical-thermal characteristics of silicon-particle anodes, exploring the effects of temperature, charging rates, and lithiation/de-lithiation cycles, and validated the theoretical results with experimental results. Li et al.^35^ developed an anisotropic chemo-mechanical model with anisotropic properties of crystalline silicon and analyzed deformation and stress fields in Li_15_Si_4_. Their results demonstrate that anisotropic expansion could potentially reduce von Mises stress in [110]-oriented silicon nanopillars, which could retard mechanical degradation of silicon-based electrodes. Bhowmick and Chakraborty^36^ included orientation-dependent interface rate constants in the analysis of the lithium migration across the interface between a lithium-rich amorphous silicide and a crystalline silicon under the framework of finite deformation and revealed nonuniform distributions in lithium concentration and chemical stress as well as the evolution of facets at the crystalline-amorphous interface during the initial lithiation of a crystalline silicon cylinder. Wang et al.^37^ suggested that the fracture of electrode during lithium-insertion is problematic due to combinational effects of surface stresses, plastic deformation, and strain gradient effects and showed that surface modification can likely enhance structural integrity of electrodes and reduce surface stresses.

Various approaches have been proposed to mitigate lithium trapping, such as developing protective coatings, improving the conductivity of electrolyte, and modifying electrode designs.^38^^,^^39^^,^^40^ However, there are few works addressing the effects of lithium trapping on the evolution of chemical stress during electrochemical cycling. Lithium trapping generally is an irreversible process, which can result in a permanent loss of lithium in electrolyte and a separation of lithium-rich phases from electrode.^41^^,^^42^ The phenomenon, referred to as "dead Li", has been observed in isolated lithium metal structures and particles that are no longer in contact with the current collector.^41^ This work presents a finite deformation model that incorporates the effect of lithium trapping on stress evolution and mass transport. Solute atoms (lithium) are categorized into mobile and immobile solute atoms: mobile solute atoms are associated with diffusion, and immobile solute atoms are associated with chemical reactions and lithium trapping. The proposed finite deformation model can likely provide more insight into the effects of lithium trapping on the stress evolution and mass transport in lithium-ion batteries and can be used to investigate the deformation behavior of electrodes under various cycling conditions. The results can help to develop effective strategies to mitigate lithium trapping and to reduce the capacity loss associated with lithium trapping. It needs to be pointed out that the analysis in this work does not discuss the interaction between trapped lithium and plastic deformation. However, the approach used in this work can be extended to analyze the effects of trapped lithium on visco-plastic deformation presented in electrode materials of similar systems, such as Sn or Ge electrodes.^43^^,^^44^

### Model establishment

#### Lithium trapping during electrochemical cycling

Rehnlund et al.^17^ suggested that lithium trapping is associated with lithium diffusion in electrode and used a two-way diffusion trapping model in explaining the phenomenon of lithium trapping. They introduced the concept of “dead lithium”, which is isolated from electrode and does not participate in further cycling process. This work does not consider the contribution of the “dead lithium” to the trapping of lithium and is focused on the effects of the dissociation of intermetallic compounds (alloys).

In general, the diffusion of metal atoms in electrodes of a metal-ion battery can lead to the formation and dissociation of chemical compounds.^45^^,^^46^ For example, the lithiation of lithium-ion batteries with carbon and silicon as anode can result in the formation of Li_x_C and Li_x_Si,^47^^,^^48^ respectively, which is also referred to as the intercalation reaction. Mathematically, a reaction-diffusion system can be described by a parabolic partial differential equation. Taking into account the effect of stress on diffusion, the diffusion equation in a largely deformed solid can be formulated as^44^

(Equation 1) $$D_{t}C=\mathrm{Div}\left(\frac{DC}{R_{g}T}F^{-1}F^{-T}Grad\mu \right)-R_{s}\text{.}$$

Here, *D*_t_ represents the material time derivative and can be calculated as ∂/∂t+**v**·grad in the current configuration with the velocity field **v** and gradient operator “grad” in the current configuration, *C* represents the concentration of diffusive atoms (mobile atoms) in the initial configuration, *D* represents the diffusion coefficient (which is constant in a homogeneous solid), **F** represents the deformation gradient tensor, “Grad” represents the gradient operator in the initial configuration, *μ* represents the chemical potential of the diffusive specie, and *R*_*s*_ is the production rate for local solid reactions. The chemical potential of the diffusive atoms in a stress field for an ideal solution is expressed as^49^

(Equation 2) $$\mu =\mu_{0}+R_{g}T\;\ln\;c-\Omega_{1}\sigma_{h}+\Omega_{2}w\text{,}$$

where μ_0_ is the chemical potential of the diffusive specie at the standard state without stress field, *c* is the concentration of diffusive atoms in the current configuration and is correlated to *C* as *C* = det(**F**)*c*, Ω_1_ is the volumetric expansion coefficient per mole of the diffusive atoms, Ω_2_ is the molar volume of the host material, and *w* is the strain energy density in the current configuration.

During insertion, a local solid reaction of *aA* + *bB*→*fF* occurs. The reaction rate for the chemical product, *R*_*s*_, is

(Equation 3) $${R_{s}|}_{lithiation}=D_{t}S=kC^{a}C_{2}^{b}\text{,}$$

where *S* is the concentration of immobile solute atoms due to the local solid reaction with host atoms, such as the formation of silicide for silicon-based electrodes, *k* is the forward reaction rate constant, *C*_2_ is the concentration of the host material, and *a* and *b* are constants. During de-insertion, local solid reaction of *fF*→*aA* + *bB* occurs. The dissociation rate, *R*_*s*_, (de-alloying) is assumed to be proportional to the concentration of immobile solute atoms as^50^

(Equation 4) $${R_{s}|}_{de-lithiation}=D_{t}S=-\lambda S\text{,}$$

where *λ* is the backward reaction rate constant. In general, it is impossible to have complete dissociation of the chemical compounds during de-insertion, which leads to the trapping of diffusive atoms, such as lithium trapping. Note that the mobile solute atoms are referred to the solute atoms, which are not pinned in the host material and movable under the action of a driving force, such as the gradient of chemical potential. The immobile solute atoms are referred to the solute atoms, which are pinned in the host material and immovable under the action of a driving force. In this work, the pinning mechanism is attributed to the formation of chemical compounds, which eventually leads to the trapping of solute atoms.

The trapping effect in lithium-ion batteries is referred to the phenomenon where a portion of lithium atoms becomes trapped and immobile within the host material during electrochemical cycling. The trapped lithium is unable to further participate in the storage of electrochemical energy that occurs during the battery operation, resulting in the loss of capacity. There are various factors likely contributing to the trapping effect, including the formation of a passivating layer^51^^,^^52^ and the phase evolution of active material^41^^,^^53^ during the battery operation. For example, during charging, lithium ions migrate from cathode to anode, where they form chemical compounds in the anode. During discharging, some of the chemical compounds formed during the charging remain unchanged, which cannot revert back to their initial state. This trend leaves behind residual compounds with trapped lithium.

It should be noted that the lithium trapping discussed in this work is due to a difference in the driving force between the forward reaction and the backward reaction. For example, there generally exists a difference in the driving force for the forward reaction of *aA* + *bB*→*fF* and the backward reaction of *fF*→*aA* + *bB*. Assuming that the free energy of *fF* is larger than the total free energy of *A* and *B*, an external force, such as electromotive force, is required to activate the forward reaction, and no external force is needed for the start of the backward reaction once the external force is removed. The backward reaction will continue until the free energy of the system reaches a minimum. The minimum free energy is dependent on the energy barrier for the backward reaction and corresponds to an equilibrium state between the reactants and the product. This hinders a complete reverse of *A* and *B* from *F* and results in the loss of *A* and *B* (trapped lithium in this work).

**Figure 1:**
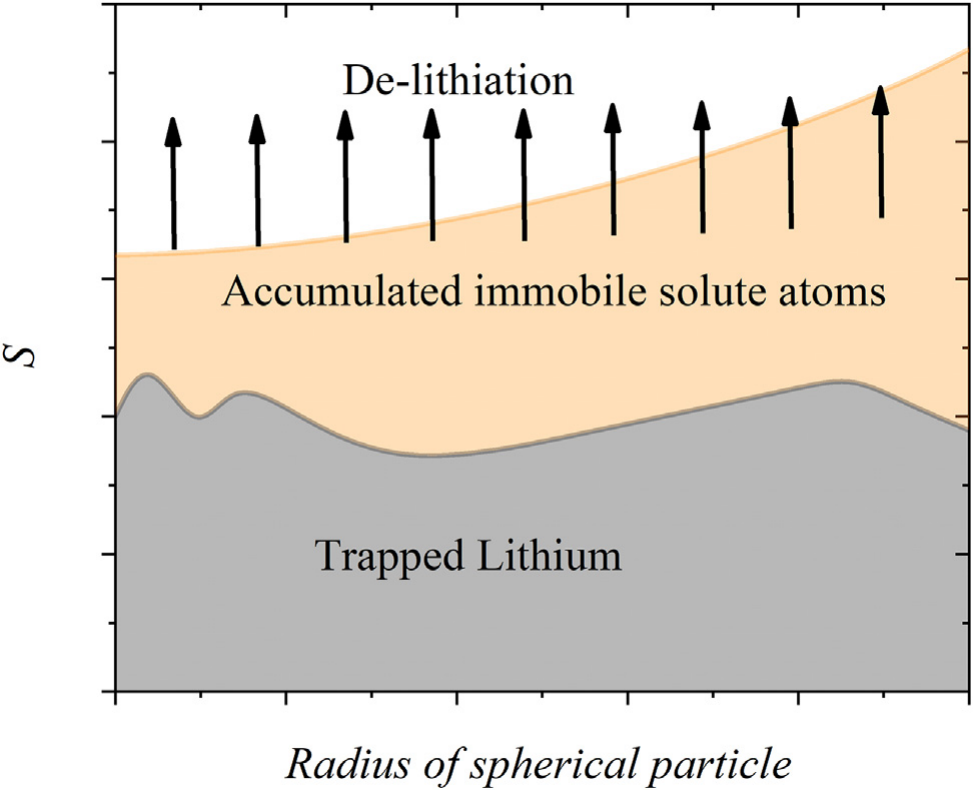
Schematic of lithium trapping during de-lithiation During the lithiation process, the concentration of immobile lithium, denoted as *S*, increases; during de-lithiation, the reaction product associated with immobile lithium decomposes, leading to the release of immobile lithium. The reaction product will not likely decompose completely, resulting in a fraction of lithium being trapped within the host material.

Assume that the trapped lithium in a lithium-ion battery is only related to immobile lithium. Figure 1 schematically illustrates this phenomenon. During lithiation, the concentration of immobile lithium, *S*, increases, which is due to the formation of the reaction product. During de-lithiation, it is unlikely for the reaction product to decompose completely to release all the immobile lithium. A portion of lithium is trapped within the host material,^54^ as depicted in Figure 1. Denote *C*_trap_(*X*) as the residual concentration of the trapped lithium, which is a spatial function. Thus, the reaction rate *R*_*s*_ in Equation 4 for the de-lithiation can be expressed as

(Equation 5) $${R_{s}|}_{de-lithiation}=D_{t}S=\left\{ \begin{array}{c} \begin{array}{cc} -\lambda S & \begin{array}{cc} for & S>C_{trap} \end{array} \end{array}\\ \begin{array}{cc} 0 & \begin{array}{cc} for & S\leq C_{trap} \end{array} \end{array} \end{array}\right.\text{.}$$

The physical significance of Equation 5 indicates that the reaction product remains unchanged for *S* ≤ *C*_trap_. For example, if we set the residual concentration of trapped lithium as constant, the concentration of the trapped lithium at the end of “complete” de-lithiation is constant everywhere in the electrode. It needs to be pointed out that the concentration of immobile lithium, *S*, defined in this work is different from the one used by Rehnlund et al.*,*^17^ who referred trapped lithium to as immobile lithium in the analysis of "dead lithium" when a portion of the active material with lithium is broken off and electrically disconnected from the electrode. Here, the immobile lithium consists of two portions: one is from the reaction product, the other is from the trapped lithium, as illustrated in Figure 1.

Under potentiostatic operation, where a constant concentration *C*_max_ is maintained at the surface of a spherical electrode with *R*_0_ as the initial radius, the initial and boundary conditions for an electrode, which is initially devoid of lithium, can be formulated as follows.

(Equation 6) $$C\left(R,0\right)=0,\text{for}\;0\leq R\leq R_{0}\text{,}$$

$$C\left(R_{0},t\right)=C_{\max},\text{for}\;\text{lithiation}\;\text{process}$$

$$C\left(R_{0},t\right)=0,\text{for}\;\text{de}-\text{lithiation}\;\text{process}$$

C(0,t)=finite,fort≥0.

The concentrations of mobile lithium *C* and immobile lithium *S* can be calculated from Equations 1, 2, 3, 4, and 5 with the conditions of 6.

#### Stress and strain in a largely deformed spherical electrode

Consider an isotropic spherical particle. The coordinate system is denoted as (*R*, *Θ*, *Φ*) in the initial configuration and (*r*, *θ*, *φ*) in the current configuration. The non-zero radial displacement, *u*, is calculated as *u* = *r*－*R*. The radial and hoop components of the total deformation gradient tensor are *F*_*R*_ = 1+∂*u*/∂*R*, and *F*_*Θ*_ = *F*_*Φ*_ = 1+∂*u*/∂*R*. Using a quadratic function of the Green-Lagrange strain tensor to represent the strain energy density within the framework of the Lagrangian description, we have

(Equation 7) $$W=\frac{\det\left(F^{c}\right)E_{h}}{2\left(1+\nu \right)}\left[\frac{\nu }{\left(1-2\nu \right)}{\left[tr\left(E^{e}\right)\right]}^{2}+tr\left(E^{e}E^{e}\right)\right]\text{.}$$

It should be noted that the strain energy density, *w*, within the framework of the Euler description in Equation 2 is associated with the counterpart, *W*, within the framework of the Lagrangian description. Following the work of Cui et al. [26], the constitutive relation between the first Piola-Kirchoff stress tensor **Pk** for a largely deformed solid without plastic deformation is calculated as

(Equation 8) $$Pk=\frac{\det\left(F^{c}\right)E_{h}}{\left(1+\nu \right)}F^{e}\left[\frac{\nu }{\left(1-2\nu \right)}tr\left(E^{e}\right)I+E^{e}\right]{\left(F^{c}\right)}^{-T}\text{,}$$

where **F**^e^ and **F**^c^ are the elastic and eigen-transformation deformation gradient tensors, respectively. These deformation gradient tensors are interconnected with the total deformation gradient tensor **F** through the equation **F**= **F**^e^**F**^c^. Note that the first Piola-Kirchoff stress tensor **Pk** is related to the Cauchy stress tensor **σ** as **σ** = det^−1^(**F**)**PkF**^T^. For isotropic deformation, the eigen-transformation deformation gradient tensor can be calculated as^50^

(Equation 9) $$F_{R}^{c}=F_{\Theta }^{c}=F_{\Phi }^{c}={\left(1+\Omega_{1}C+\varpi S\right)}^{1/3}\text{.}$$

Here, ϖ denotes the volumetric change per mole of immobile lithium. The physical significance of Equation 9 lies in the fact that the electrode deformation depends not only on the concentration of mobile lithium, *C*, but also on the concentration of immobile lithium, *S*. The elastic component of the Green Lagrangian strain tensor **E**^e^ is calculated as

(Equation 10) $$E^{e}=\frac{1}{2}\left({\left(F^{e}\right)}^{T}F^{e}-I\right)\text{,}$$

Substituting Equation 9 in **F**= **F**^e^**F**^c^ and using *F*_*R*_ = 1+∂*u*/∂*R*, and *F*_*Θ*_ = *F*_*Φ*_ = 1+∂*u*/∂*R*, we obtain from Equation 10 the following relationships.

(Equation 11) $$E_{R}^{e}=\frac{1}{2}\left[{\left(1+\Omega_{1}C+\varpi S\right)}^{-2/3}{\left(1+\partial u/\partial R\right)}^{2}-1\right]\text{,}$$

(Equation 12) $$E_{\Theta }^{e}=E_{\Phi }^{e}=\frac{1}{2}\left[{\left(1+\Omega_{1}C+\varpi S\right)}^{-2/3}{\left(1+u/R\right)}^{2}-1\right]\text{.}$$

The radial and tangential components of the first Piola-Kirchhoff stress tensor are calculated from Equations 8, 9, 10, 11, and 12 as

(Equation 13) $$Pk_{R}=\frac{\left(1+\Omega_{1}C+\varpi S\right)E_{h}}{\left(1+\nu \right)\left(1-2\nu \right)}\left[\left(1-\nu \right)E_{R}^{e}+2\nu E_{\Theta }^{e}\right]\frac{{\left(F_{R}^{e}\right)}^{2}}{F_{R}}\text{,}$$

(Equation 14) $$Pk_{\Theta }=Pk_{\Phi }=\frac{\left(1+\Omega_{1}C+\varpi S\right)E_{h}}{\left(1+\nu \right)\left(1-2\nu \right)}\left[\nu E_{R}^{e}+E_{\Theta }^{e}\right]\frac{{\left(F_{\Theta }^{e}\right)}^{2}}{F_{\Theta }}\text{.}$$

The equilibrium equations governing mechanical deformation within an arbitrary domain for the cycling-induced deformation of the spherical electrode can be expressed as

(Equation 15) $$\frac{\partial Pk_{R}}{\partial R}+2\frac{Pk_{R}-Pk_{\Theta }}{R}=0\text{,}$$

With the characteristic of spherical symmetry, the boundary conditions without surface traction are *u*(*R* = 0) = 0 and *Pk*_*R*_(*R* = *R*_0_) = 0. It is worth mentioning that although the analysis of the lithiation process in this work is similar to some works reported in the literature,^44^ the de-lithiation process with the trapping effect has not been reported in the literature.

## Results

### Numerical implementation and results

An amorphous spherical silicon nanoparticle is used in the numerical calculation to explore the effects of lithium trapping on stress evolution and mass transport. The coupled equations are solved numerically with the finite element module of COMSOL Multiphysics software. The finite element model used in this work consists of 1,000 2nd order elements, ensuring a sufficiently fine mesh to capture the complex deformation behavior and mass transport. To have a high accuracy of the numerical results, a convergence tolerance of 1 × 10^−9^ is used.

Table 1 lists the parameters used in the numerical calculation. It is expected that the stress evolution and mass transport within the spherical silicon nanoparticle are significantly dependent on the forward reaction rate constant *k* in Equation 3 and the backward reaction rate constant *λ* in Equation 5. According to the work by Li et al.,^44^ we set $kC_{2}^{b}$ to be 0.01. The backward reaction rate constant *λ* is assumed to be 0.05. Two specific cases are considered. The first one uses a constant residual concentration of trapped lithium, and the second one considers spatial dependence of the residual concentration of trapped lithium.

**Table 1:** Properties of Si and parameters used in the numerical calculation

Property/Parameter	Symbol	Value
Young’s Modulus of pristine silicon	*E*_*h*_	90 GPa^49^
Poisson’s ratio	*ν*	0.28^49^
Volumetric expansion coefficient per mole of mobile lithium	Ω_1_	8.18 × 10^−6^ m^3^·mol^−1^^49^
Volumetric change per mole of immobile lithium	ϖ	Assumed to be the same as Ω_1_
Partial molar volume of silicon	Ω_2_	Assumed to be the same as Ω_1_
Diffusion coefficient	*D*	1 × 10^−16^ m^2^·s^−1^^49^
Maximum concentration	*C*_max_	3.67 × 10^5^ mol·m^−3^^49^
Initial radius	*R*_0_	250 nm
Gas constant	*R*_*g*_	8.3145 J·mol^−1^·K^−1^
Absolute temperature	*T*	298 K

#### Case I: Constant residual concentration of trapped lithium

**Figure 2:**
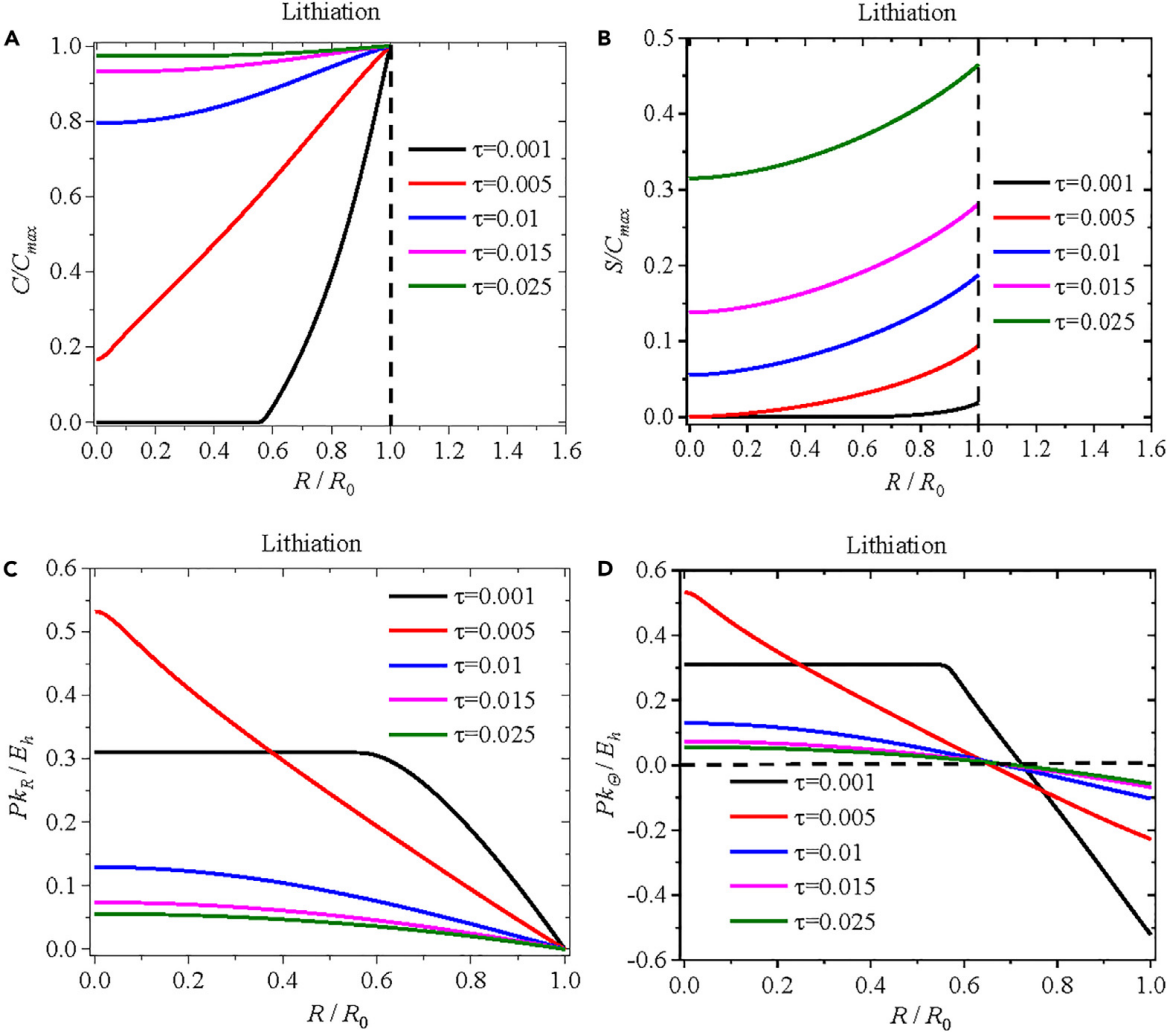
Distributions of normalized concentration of lithium and Piola-Kirchhoff stress during lithiation Spatial distributions of (A) the normalized concentration of mobile lithium, (B) the normalized concentration of immobile lithium, (C) the normalized radial component of Piola-Kirchhoff stress, and (D) the normalized hoop component of Piola-Kirchhoff stress at the lithiation times of *τ* = 0.001, 0.005, 0.01, 0.015, and 0.025 during lithiation under potentiostatic operation.

We first analyze the case with constant residual concentration of trapped lithium during electrochemical cycling. Here, we assume the residual concentration of trapped lithium to be 0.2*C*_max_. Figure 2 depicts spatial distributions of (*a*) the normalized concentration of mobile lithium, (*b*) the normalized concentration of immobile lithium, (*c*) the radial component of the Piola-Kirchhoff stress, and (*d*) the hoop component of the Piola-Kirchhoff stress at specific lithiation instants (*τ = Dt/R*_0_^2^ = 0.001, 0.005, 0.01, 0.015, 0.025) during lithiation under the potentiostatic operation. According to Figure 2A, the normalized concentration of mobile lithium at the surface of the spherical silicon electrode remains constant, as expected, during the lithiation. The concentration of mobile lithium at the center of the spherical silicon electrode continuously increases, reaching a peak value at a normalized time of τ = 0.025. It needs to be pointed out that the analysis does not include a dwell period to allow the concentration of mobile lithium to reach a steady state. Therefore, the lithiation state at the normalized time τ = 0.025 is regarded as the final state of the lithiation process, which also corresponds to the onset of de-lithiation.

According to Figure 2B, the concentration of immobile lithium at the same position continuously increases with increasing the lithiation time. This result indicates continuous increase of the chemical compounds formed from chemical reactions during the lithiation. The largest concentration of immobile lithium is present at the surface of the electrode, as expected.

The spatiotemporal evolutions of the stress components shown in Figures 2C and 2D reveal that both the radial and hoop components of the Piola-Kirchhoff stress at the center of the spherical electrode are tensile. The magnitudes of both the radial and hoop components of the Piola-Kirchhoff stress at the center of the spherical electrode increase initially, decrease subsequently, and approach zero eventually. At the surface of the spherical electrode, the radial component of the Piola-Kirchhoff stress is zero, as expected from the traction-free condition. The hoop component of the Piola-Kirchhoff stress at the surface of the spherical electrode is compressive, and its magnitude reaches maximum at the early stage of the lithiation and gradually decreases with the increase of the lithiation time. Such a trend is similar to the one given by Yang^55^ and suggests that the chemical stress within the spherical electrode is primarily controlled by the concentration gradient of lithium. Thus, both the mobile lithium and immobile lithium are expected to contribute to the evolution of the eigen-strain, thereby leading to significant stress levels in the spherical electrode during the lithiation.

**Figure 3:**
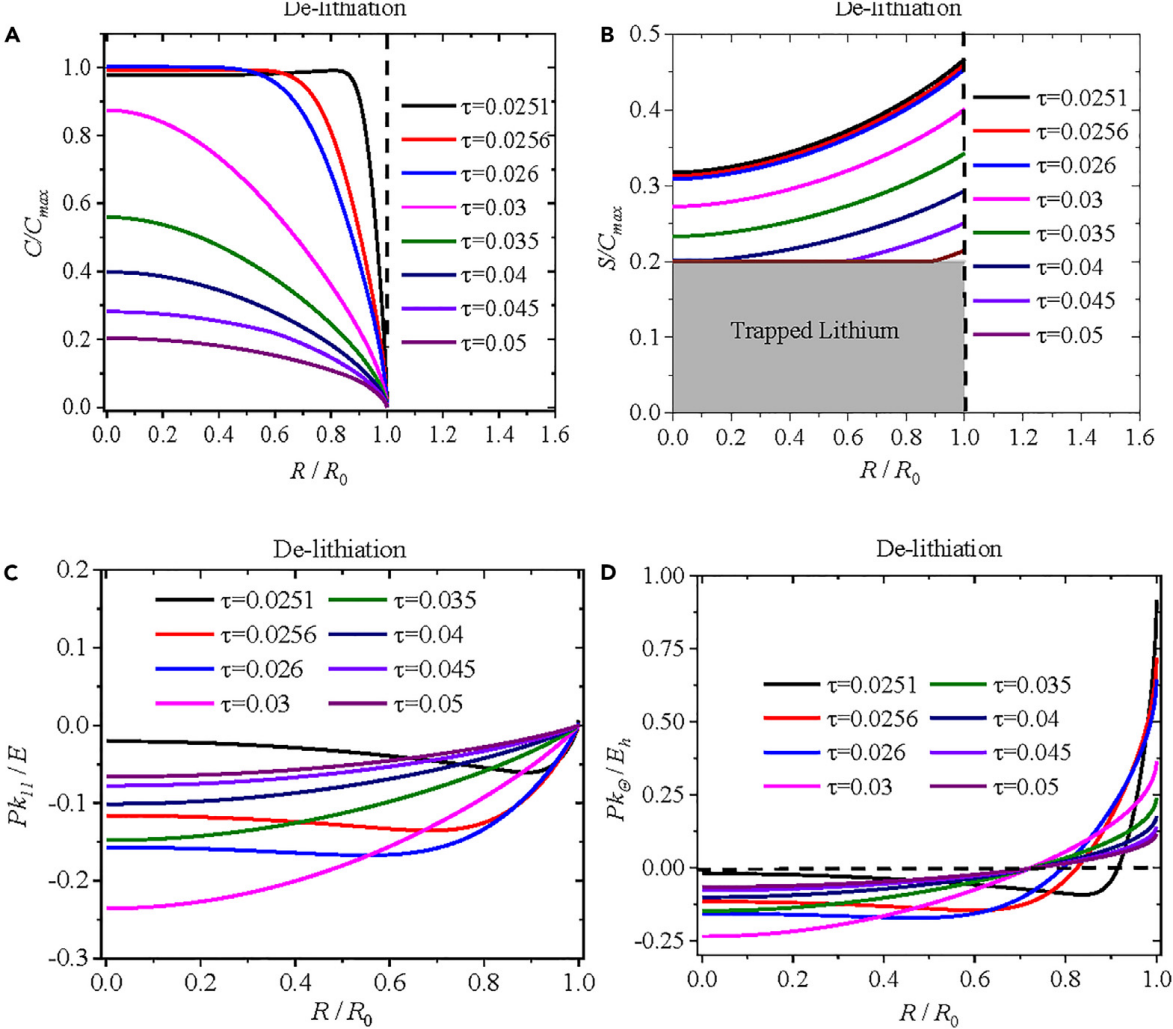
Distributions of normalized concentration of lithium and Piola-Kirchhoff stress during de-lithiation Spatial distributions of (A) the normalized concentration of mobile lithium, (B) the normalized concentration of immobile lithium, (C) the normalized radial component of Piola-Kirchhoff stress, and (D) the normalized hoop component of Piola-Kirchhoff stress at the cycling times of *τ* = 0.0251, 0.0256, 0.026, 0.03, 0.035, 0.04, 0.045, and 0.05 during de-lithiation under potentiostatic operation.

Figure 3 presents spatial distributions of (*a*) the normalized concentration of mobile lithium, (*b*) the normalized concentration of immobile lithium, (*c*) the radial component of the Piola-Kirchhoff stress, and (*d*) the hoop component of the Piola-Kirchhoff stress at different cycling times (τ = 0.0251, 0.0256, 0.026, 0.03, 0.035, 0.04, 0.045, 0.05) during de-lithiation under potentiostatic operation. From Figure 3A, we note that the normalized concentration of mobile lithium at the surface of the spherical electrode remains zero during the de-lithiation, as required by the boundary condition. Notably, the normalized concentration of mobile lithium at the center of the spherical electrode increases for the normalized cycling time less than or equal to 0.026 and then decreases with the increase of the normalized cycling time to 0.05. This behavior can be attributed to the presence of a small concentration gradient of mobile lithium at the end of the lithiation, corresponding to the normalized cycling time of τ = 0.025, which continuously drives lithium into the spherical electrode. According to Figure 3B, the normalized concentration of immobile lithium decreases gradually with the increase of the de-lithiation time. However, the concentration of immobile lithium stops decreasing when the normalized concentration of immobile lithium *S*/*C*_max_ reaches the critical value of 0.2 for trapped lithium. Afterward, the size with the critical concentration of 0.2*C*_max_ for trapped lithium increases with the increase of the de-lithiation time. It is noteworthy mentioning that the direction of the concentration gradient of immobile lithium is different from that of mobile lithium during the de-lithiation. Such a difference implies that a small concentration gradient of immobile lithium may introduce a large chemical stress during the de-lithiation.

The spatiotemporal evolution of the normalized radial and hoop components of the Piola-Kirchhoff stress is presented in Figures 3C and 3D. The spherical electrode experiences compressive stress in the radial direction during the de-lithiation. The magnitude of the compressive stress at the center of the spherical electrode increases first, reaches maximum, and then decreases to zero with the increase of the de-lithiation time. At the surface of the spherical electrode, the radial component of the Piola-Kirchhoff stress remains zero, as required by the traction-free condition. The spherical electrode experiences compressive stress around the center of the electrode and tensile stress near the surface of the electrode in the hoop direction. The size of the compressive stress zone decreases with the increase of the de-lithiation time and reaches constant for the cycling time larger than or equal to 0.03. The maximum hoop component of the Piola-Kirchhoff stress is present at the surface of the electrode, and the magnitude of the maximum hoop component decreases with increase of the de-lithiation time. Note that both the radial and hoop components of the Piola-Kirchhoff stress depict an S-shaped pattern at the dimensionless time τ = 0.0251. This behavior can be attributed to the contribution of mobile lithium, as shown in Figure 3A for the spatial distribution of the concentration of mobile lithium, which is caused by an abrupt change from the lithiation to the de-lithiation and slightly S-shaped at normalized times of 0.0251, 0.0256, and 0.026.

**Figure 4:**
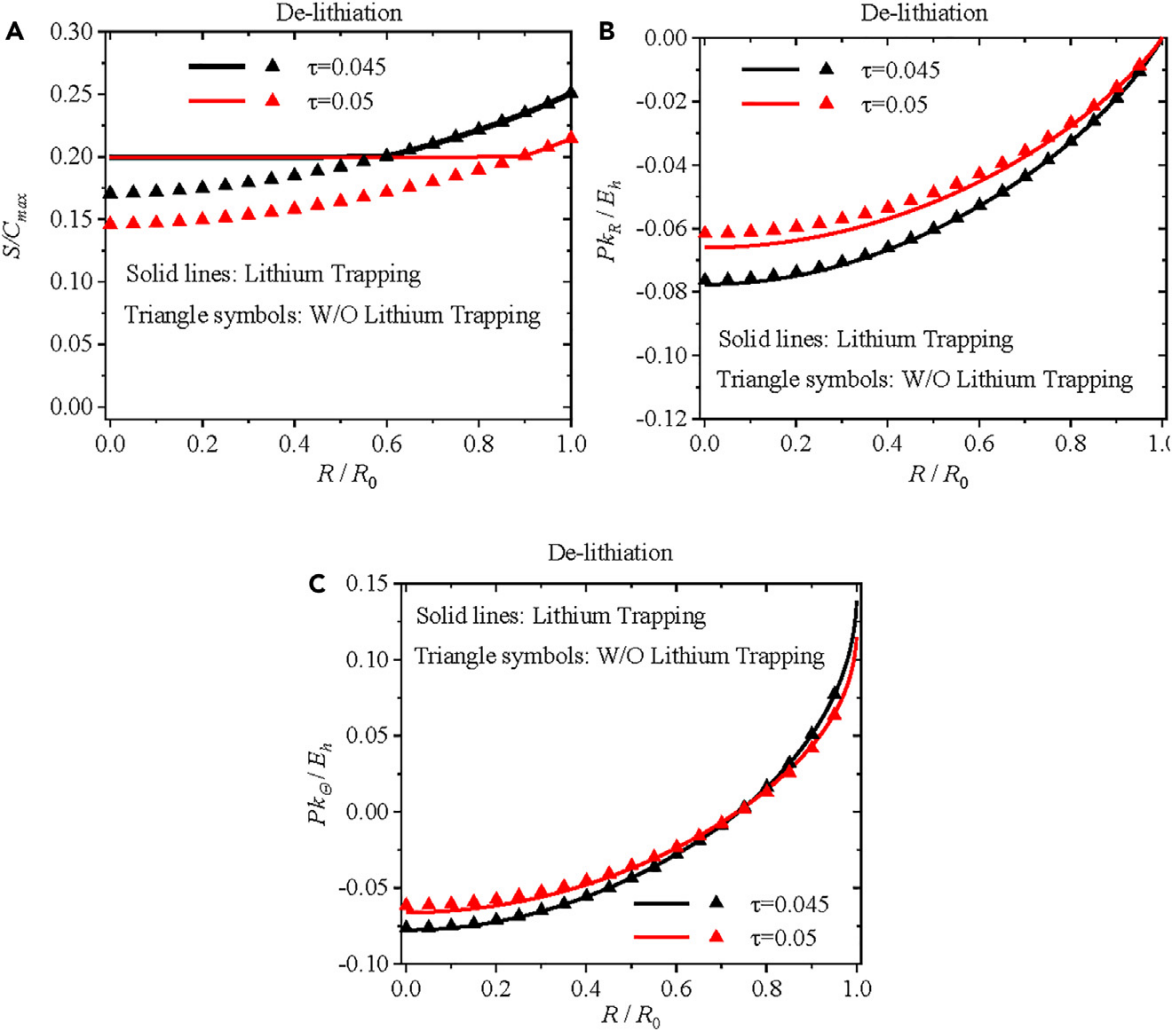
Comparison of numerical results with and without the lithium trapping Comparison of the numerical results with and without the lithium trapping at the normalized cycling times of *τ* = 0.045 and 0.05 during de-lithiation under potentiostatic operation: (A) the normalized concentration of immobile lithium, (B) the normalized radial component of Piola-Kirchhoff stress, and (C) the normalized hoop component of Piola-Kirchhoff stress. Solid lines represent the numerical results with lithium trapping, and triangle symbols represent the numerical results without lithium trapping.

From the aforementioned numerical results, we can conclude that lithium trapping has a significant effect on the evolution of chemical stress associated with the concentration gradient of immobile lithium. To illustrate the influence of lithium trapping on the migration of lithium and the evolution of chemical stress, we present the comparison of the numerical results with and without the lithium trapping in Figure 4. The solid lines represent the numerical results with the lithium trapping, and the triangle symbols represent the numerical results without the lithium trapping. Here, numerical values of 0 and 0.2C_max_ are used for *C*_trap_ in the numerical calculation, corresponding to the cases without and with the lithium trapping, respectively.

Figure 4A depicts the comparison of the normalized concentration of immobile lithium with and without the lithium trapping. For *S* larger than 0.2C_max_, the triangle symbols nearly overlap with the corresponding solid lines at τ = 0.045 and 0.05; for *S* less than 0.2C_max_, the triangle symbols deviate from the corresponding solid lines at the same spatial position at τ = 0.045 and 0.05. Thus, the concentration gradient of the normalized immobile lithium with the lithium trapping is smaller than that without the trapping effect, thereby leading to large chemical stresses, as revealed in Figures 4B and 4C. The magnitudes of both the compressive radial and hoop components of the Piola-Kirchhoff stress with the lithium trapping are slightly larger than the corresponding ones without the lithium trapping at the same spatial position. This result confirms that the lithium trapping plays a role in the evolution of the Piola-Kirchhoff stress during the de-lithiation.

**Figure 5:**
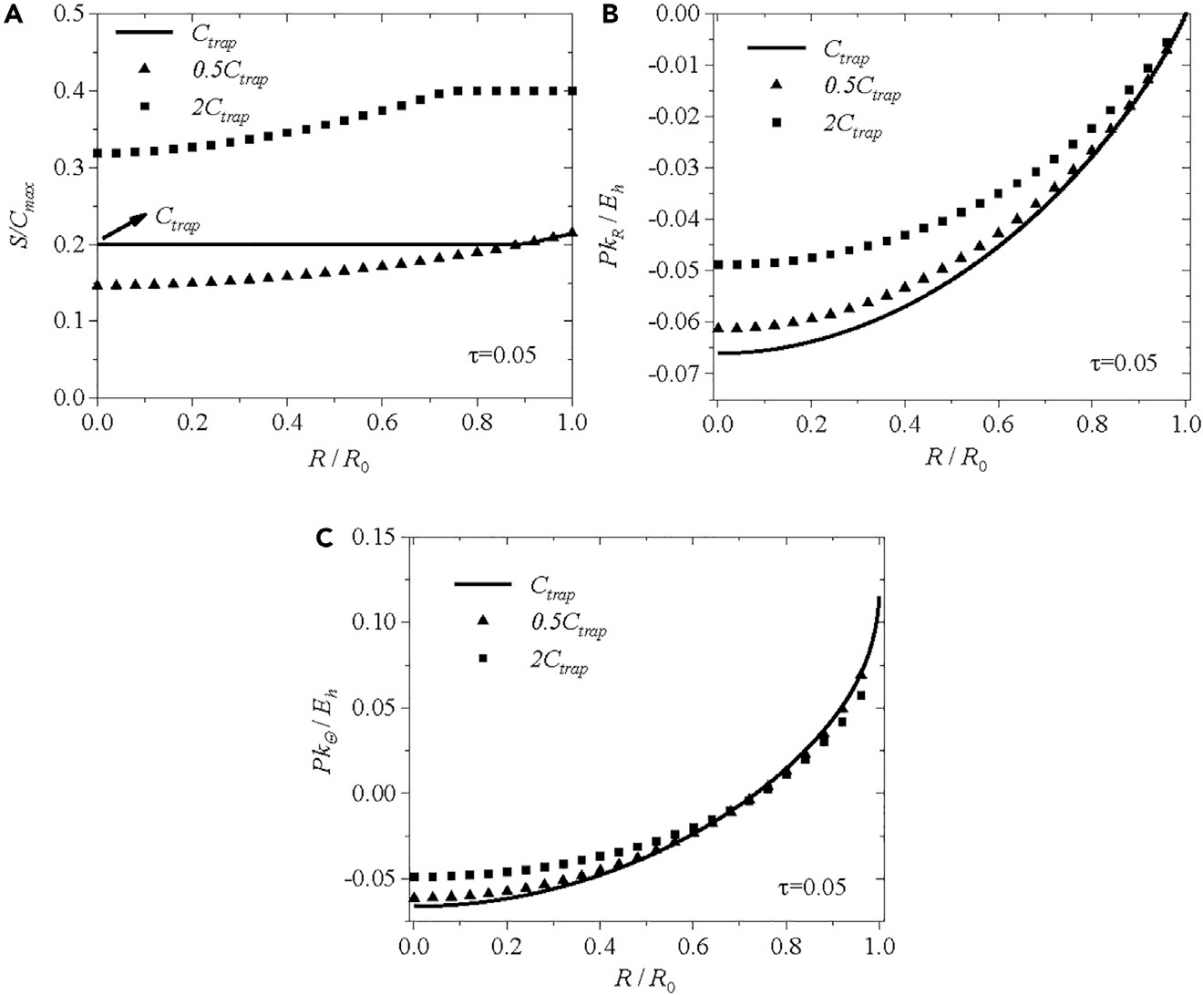
Comparison of numerical results for three residual concentrations of trapped lithium Comparison of the numerical results for three different residual concentrations of 0.1*C*_max_ (0.5*C*_trap_), 0.2*C*_max_ (*C*_trap_), and 0.4*C*_max_ (2*C*_trap_) for trapped lithium at *τ* = 0.05 during de-lithiation under potentiostatic operation: (A) the normalized concentration of immobile lithium, (B) the normalized radial component of Piola-Kirchhoff stress, and (C) the normalized hoop component of Piola-Kirchhoff stress.

To further explore the impact of lithium trapping on stress evolution and mass transport of mobile and immobile lithium, we consider three different residual concentrations of 0.1*C*_max_ (0.5*C*_trap_), 0.2*C*_max_ (*C*_trap_), and 0.4*C*_max_ (2*C*_trap_) for trapped lithium, as illustrated in Figure 5. According to Figure 5A, the immobile lithium is trapped in the inner core of the spherical electrode for the residual concentrations of trapped lithium being 2*C*_trap_ (0.4*C*_max_) at the normalized time τ = 0.05 and becomes unable to diffuse out of the electrode during the de-lithiation. This distribution of the concentration of immobile lithium results in a large concentration gradient and subsequently leads to small magnitudes of the radial and hoop components of the Piola-Kirchhoff stress at the electrode center, as supported by the stress distributions shown in Figures 5B and 5C. For the residual concentrations of trapped lithium being 0.5*C*_trap_ (0.1*C*_max_), the constraint of *R*_s_|_de-lithiation_ = 0 for *S* < *C*_trap_ is inapplicable. More immobile lithium is released from the reaction product and can diffuse out the electrode at the dimensionless times of τ = 0.05. In this scenario, the distribution of the concentration of immobile lithium at τ = 0.05 is the same as that without the lithium trapping. Note that the concentration gradient of immobile lithium with 0.5*C*_trap_ is greater than that with *C*_trap_ and smaller than that with 2*C*_trap_. Thus, the magnitudes of the compressive radial and hoop components of the Piola-Kirchhoff stress are smallest for the case with 2*C*_trap_, followed by the case with 0.5*C*_trap_, and largest with *C*_trap_, as shown in Figures 5B and 5C. The difference of the stresses with and without the lithium trapping increases with the increase of the residual concentration of trapped lithium.

#### Case II: Inhomogeneous residual concentration of trapped lithium

In the above analysis, we assume a constant residual lithium concentration of 0.2C_max_ for the lithium trapping.^17^^,^^56^ Such a constant residual concentration for trapped lithium may not be the sole situation presented in lithium-ion batteries during electrochemical operation. According to experimental results, the distribution of trapped lithium within nanoparticles may exhibit a nonuniform pattern. Also, it is very difficult, if not impossible, to experimentally obtain the distribution of the residual concentration of trapped lithium. Since more trapped lithium is more likely to be present in the regions with relatively high concentrations of immobile lithium, we assume that the residual concentration of trapped lithium is 80% of the maximum concentration of immobile lithium, i.e., *S*|_*τ*=0.025_, in the following analysis.

**Figure 6:**
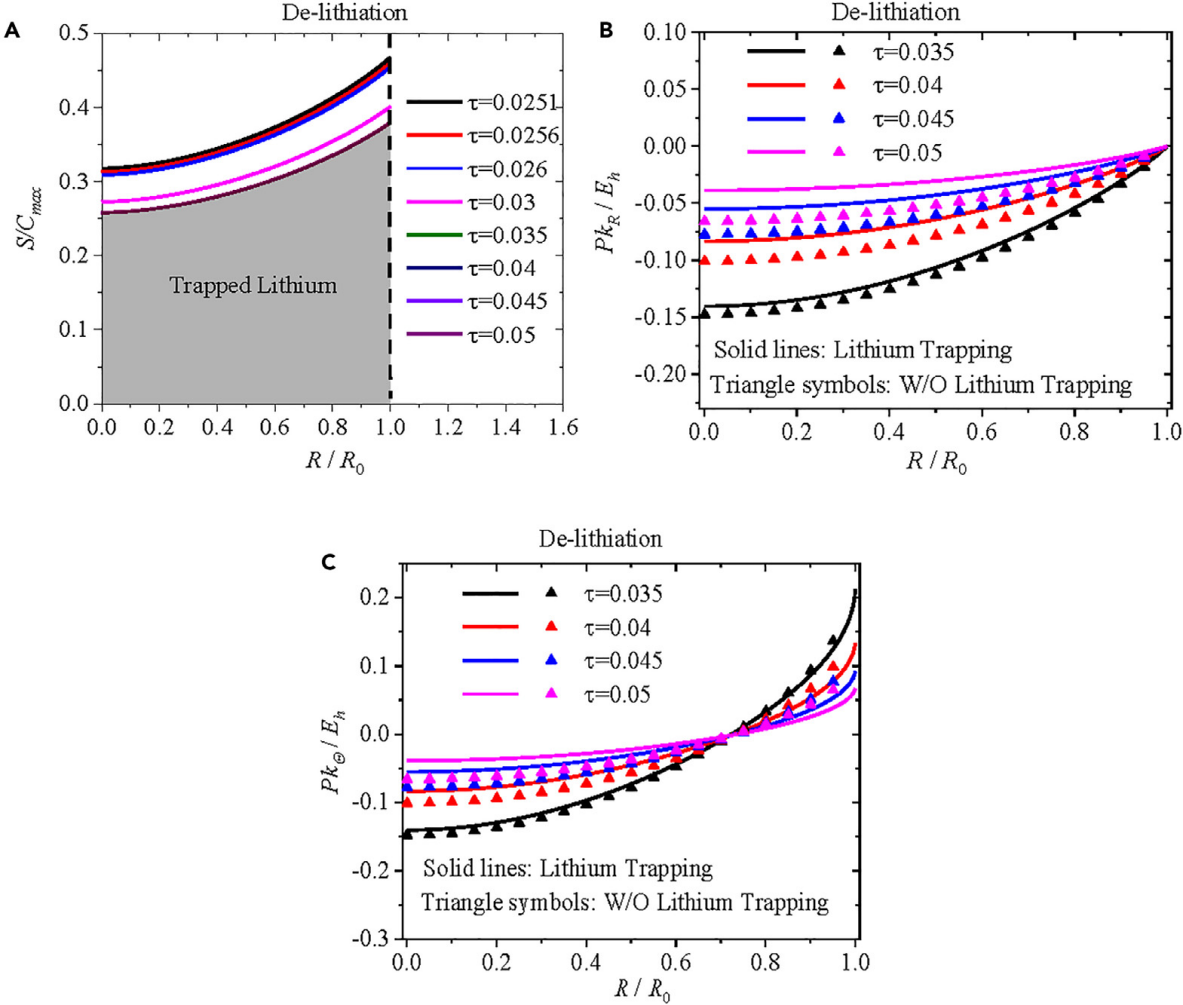
Effects of inhomogeneous residual concentration of trapped lithium on distributions of normalized concentration of lithium and Piola-Kirchhoff stress Effects of inhomogeneous residual concentration of trapped lithium on spatial distributions of (A) the normalized concentration of immobile lithium, (B) the normalized radial component of Piola-Kirchhoff stress, and (C) the normalized hoop component of Piola-Kirchhoff stress at *τ* = 0.035, 0.04, 0.045, and 0.05 during de-lithiation under potentiostatic operation. Solid lines represent the numerical results with the lithium trapping, and triangle symbols represent the numerical results without the lithium trapping.

Figure 6A presents spatial distribution of the normalized concentration of immobile lithium at τ = 0.0251, 0.0256, 0.026, 0.03, 0.035, 0.04, 0.045, and 0.05. Note that the concentrations of mobile and immobile lithium during the lithiation are not presented here since the lithium trapping has no effect on the first lithiation process. It is interesting to note from Figure 6A that the solid lines for τ = 0.035, 0.04, 0.045, and 0.05 nearly overlap, suggesting that immobile lithium stops diffusing for the normalized time larger than or equal to 0.035. This behavior is due to the fact that the residual concentration of trapped lithium is 80% of the maximum concentration of immobile lithium.

Figures 6B and 6C show spatial distributions of the radial and hoop components of the Piola-Kirchhoff stress, respectively, at τ = 0.035, 0.04, 0.045, and 0.05 with and without the lithium trapping during the de-lithiation under potentiostatic operation. The solid lines represent the numerical results with the lithium trapping, and the triangle symbols represent the numerical results without the lithium trapping. It is evident that the triangle symbols deviate from the solid lines as the de-lithiation progresses and the magnitudes of the radial and hoop components of the Piola-Kirchhoff stress with the lithium trapping are greater than the corresponding ones without the lithium trapping. This result reveals that the nonuniform distribution of trapped lithium likely alleviates chemical stress during the de-lithiation, which can likely improve the structural stability of the electrode and limit the capacity loss, as supported by the experimental results that pre-lithiation can mitigate the capacity loss of silicon electrodes.^57^^,^^58^

## Discussion

It has been recognized that lithium trapping plays an important role in the capacity loss of metal-ion batteries.^17^^,^^22^^,^^25^^,^^59^^,^^60^ There are three possible processes contributing to the trapping of lithium: one is due to the lack of driving force for the diffusion of lithium out of lithiated electrode (active materials),^61^^,^^62^ one is presented in the form of intermetallic compounds (alloys)^41^^,^^60^ due to lower free energies of the intermetallic compounds than the total free energy of the reactants for the formation of the intermetallic compounds, as discussed earlier, and the other is the dead lithium accumulated in pores,^17^^,^^41^ grain boundaries,^59^ and surfaces (plating). In most cases, all three processes can occur concurrently during electrochemical cycling for consolidated electrodes made from active particles, since the electrodes are porous and electrochemical cycling can produce intermetallic compounds and also introduce nanopores.

Hüger et al.^63^ experimentally revealed nearly uniform distribution of lithium in the form of solid and condensed Li_0.3_Si compound throughout lithiated amorphous silicon films, which supports the analysis by Yang^64^ that the lithiation of silicon with local atomic ratio of lithium to silicon less than 12/7 can likely mitigate the formation of nanopores associated with the rupture of silicon-silicon bonds. The approach used by Hüger et al.^63^ likely provides an experimental avenue to analyze the roles of the diffusion of lithium, the formation of intermetallic compounds, and the formation of nanopores in the trapping of lithium and the capacity loss of lithium-ion battery.

### Conclusion remark

In summary, we have developed a chemo-mechanical model that incorporates the contribution of lithium trapping to the mass transport and diffusion-induced stress. The lithium presented in the electrode is categorized as mobile and immobile lithium. The immobile lithium due to the solid chemical reactions, such as the formation of silicide for silicon-based electrode, during electrochemical cycling contributes to the trapping of lithium, and the lithium trapping is attributed to the residual lithium trapped in electrode during de-lithiation. Both the mobile and immobile lithium contribute to the eigen-strain due to the presence of lithium under the framework of finite deformation.

In the numerical calculation, we have considered two different cases under potentiostatic operation: one with a constant residual concentration of 0.2*C*_max_ for trapped lithium and the other with 80% of the maximum concentration of immobile lithium (*S*|_*τ*= 0.025_) for the residual concentration of trapped lithium. For a constant residual concentration of trapped lithium, the numerical results suggest that the lithium trapping likely reduces the concentration gradient of immobile lithium, thereby increasing the resultant concentration gradient of mobile and immobile lithium. Consequently, an increase in the resultant concentration gradient leads to an increase in the chemical stress. For a nonuniform distribution of the residual concentration of trapped lithium, the lithium trapping amplifies the concentration gradient of immobile lithium, resulting in a decrease in the resultant concentration gradient of mobile and immobile lithium. The decrease in the resultant concentration gradient contributes to the relief of the chemical stress. This work provides valuable insights into the effects of lithium trapping and pre-lithiation on the structural integrity of lithium-ion battery during the operation of battery, and the results can be used as a guide for further study in this field.

### Limitations of the study

It is worth noting that this work is focused on the effects of lithium trapping on the interaction between elastic deformation of a solid electrode and the diffusion of lithium. In general, there are pores in an electrode and an electrode can be present in the form of porous structure through the consolidation of active materials. An electrode made from silicon, germanium, and tin electrodes likely experiences plastic deformation during electrochemical cycling. Both porous structure (pores) and plastic deformation likely play roles in the trapping of lithium. The effects of porous structure (pores) and plastic deformation on the trapping of lithium will be incorporated in more sophisticated modeling analysis in the future.

## STAR★Methods

### Key resources table

**Table:** 

REAGENT or RESOURCE	SOURCES	IDENTIFIER
**Software and algorithms**		

COMSOL 3.5	COMSOL AB	https://www.comsol.com/

### Resource availability

#### Lead contact

Further information and requests for resources should be directed to and will be fulfilled by the lead contact, Fuqian Yang (fuqian.yang@uky.edu).

#### Materials availability

No new materials were generated in this study.

### Method details

There were numerical simulations performed in this study. The numerical simulations were performed using COMSOL PDE. More details are provided in the numerical implementation of this paper.

## Data Availability

No additional data was used. This paper does not report any original code. Any additional information generated from COMSOL PDE for reanalyzing this work is available from the lead contact upon request.
